# AnEnPi: identification and annotation of analogous enzymes

**DOI:** 10.1186/1471-2105-9-544

**Published:** 2008-12-17

**Authors:** Thomas D Otto, Ana Carolina R Guimarães, Wim M Degrave, Antonio B de Miranda

**Affiliations:** 1 Laboratory for Functional Genomics and Bioinformatics, Oswaldo Cruz Institute, Fiocruz, Rio de Janeiro, Brazil; 2 Ataulpho de Paiva Foundation, Rio de Janeiro, Brazil

## Abstract

**Background:**

Enzymes are responsible for the catalysis of the biochemical reactions in metabolic pathways. Analogous enzymes are able to catalyze the same reactions, but they present no significant sequence similarity at the primary level, and possibly different tertiary structures as well. They are thought to have arisen as the result of independent evolutionary events. A detailed study of analogous enzymes may reveal new catalytic mechanisms, add information about the origin and evolution of biochemical pathways and disclose potential targets for drug development.

**Results:**

In this work, we have constructed and implemented a new approach, AnEnPi (the Analogous Enzyme Pipeline), using a combination of bioinformatics tools like BLAST, HMMer, and in-house scripts, to assist in the identification, annotation, comparison and study of analogous and homologous enzymes. The algorithm for the detection of analogy is based i) on the construction of groups of homologous enzymes and ii) on the identification of cases where a given enzymatic activity is performed by two or more proteins without significant similarity between their primary structures. We applied this approach to a dataset obtained from KEGG Comprising all annotated enzymes, which resulted in the identification of 986 EC classes where putative analogy was detected (40.5% of all EC classes). AnEnPi is of considerable value in the construction of initial datasets that can be further curated, particularly in gene and genome annotation, in studies involving molecular evolution and metabolism and in the identification of new potential drug targets.

**Conclusion:**

AnEnPi is an efficient tool for detection and annotation of analogous enzymes and other enzymes in whole genomes. It is available for academic use at:

## Background

Enzymes catalyze biochemical reactions and are classified according to the recommendations of the Nomenclature Committee of the International Union of Biochemistry [1]. Each enzymatic activity has a recommended name and an Enzyme Commission (EC) number assigned, depending on the reaction that it catalyzes [2]. For a better understanding of the metabolism of a given species it is of utmost importance to locate, identify and annotate the genes encoding such enzymatic activities. Most approaches to perform these tasks are based on sequence similarity searches, using computational tools like BLAST [3] or Hidden Markov Models (HMMer [4]) and curated databases.

However, comparisons between computational reconstructions of metabolic pathways from different organisms revealed the existence of gaps [5]. An organism can truly lack a part of a pathway, use an alternative one, or the function is present but unannotated for different reasons (for instance, genome assembly problems). Another explanation is that some of these apparent gaps might involve alternative enzymes, also known as functional analogs [6]. Such enzymes are generally believed to be the result of independent evolutionary events [7]. Some properties of analogous enzymes include its association with different phylogenetic origins, possession of distinct catalytic mechanisms and also different foldings [8]. Automated annotation approaches, normally used for preliminary gene identification and characterization, usually employ methods based on sequence similarity criteria. These may not be able to detect analogs, as these enzymes exhibit virtually no significant sequence similarity between their primary structures [9]. In some cases it is possible to use other types of data, such as the genomic context or the experimental detection of a particular enzymatic activity, to identify the genes coding for the missing activities [10].

However, most often such genes are not characterized as analogous in the accompanying annotation, for Example in public databases such as KEGG [11].

Previous work performed by other groups suggest that the fraction of enzymatic activities where multiple events of independent origin have occurred may be substantial, in the order of 25% [9]. However, to our knowledge a global survey of these events, which also has the potential to shed light on the evolution of biochemical pathways and genome organization, has not been done.

Analogous enzymes may also constitute a huge and largely untapped resource for the identification of drug targets. Strategies to find candidate genes as potential targets for drug development usually focus on parasite-specific genes and even complete biochemical pathways [12], or for structural differences between homologues. Unfortunately, due to technical limitations, the number of available 3D structures represents only a fraction of all proteins identified so far, limiting direct structural comparisons and inducing researchers to rely on the comparison of annotation data. Since analogous enzymes, which may have Substantially different foldings – a desired pre-requisite for drug development – are not annotated as such, they may be overlooked as possible candidates for drug development.

To help in the process of identification and annotation of analogous enzymes, we implemented a web based Tool named AnEnPi. It analyses and compares genomic datasets for analogous enzymes, by clustering the primary structures of enzymes with the same described activity and using a Blastp similarity raw score of 120 as cut-off [7]. This resulted in a list of clusters that reflect substantial structural differences between enzymes with the same activity but with possibly different evolutionary origins.

## Methods

AnEnPi was programmed in Perl using the CGI-interface. All clusters as well as their HMMer-models are available for download on the web page.

**Figure 1:**
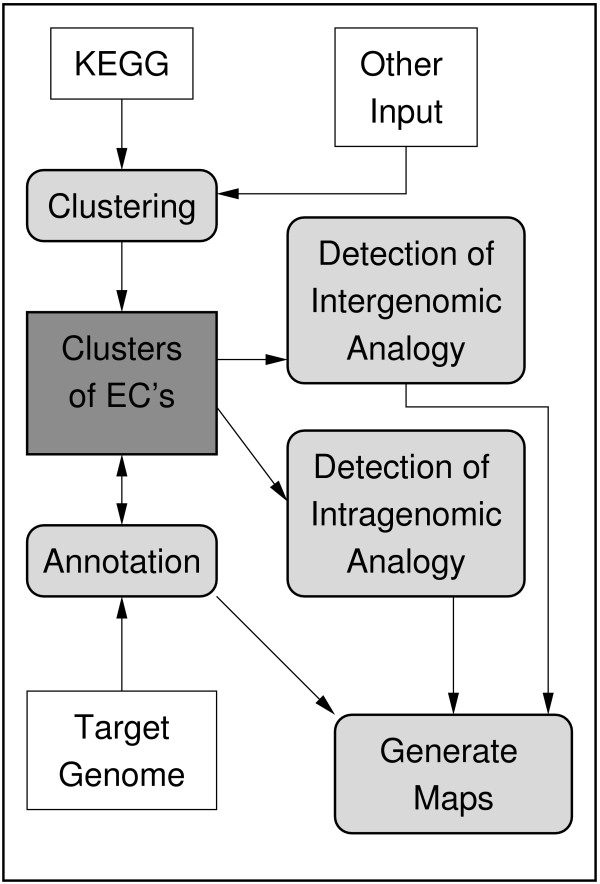
**Work flow of AnEnPi**. Databases are represented as rectangles. Darker gray rectangles represent the five datasets of clusters. Light gray rectangles are the modular functions of AnEnPi, described in the text.

An overview of AnEnPi is shown in Figure 1. For clustering we used the similarity score with a cut-off Value 120 of BLASTp pair wise comparisons between all proteins included in a specified dataset, based on the experimental work of Galperin [7]. In the work described here, groups are composed of proteins sharing the same enzymatic activity (EC classes). Within a group, protein sequences are clustered. Enzymes within a given cluster are considered homologous, while enzymes in different clusters (of the same group/function) are considered analogous. These clusters are stored in a flat file database, which can be used to annotate or re-annotate a set of proteins. To improve visualization, metabolic maps can be generated automatically.

### Dataset

We have applied AnEnPi to cluster a dataset composed of 311 reference metabolic pathways and 1,871,732 protein sequences of 36 eukaryotes, 398 eubacteria and 31 archaeabacteria obtained from the KEGG database [11,13]. In total, 326,013 sequences had a corresponding EC number assigned describing their enzymatic activity, belonging to 2,433 different EC classes. This result forms the main dataset of clusters used by AnEnPi.

### Clustering

**Figure 2:**
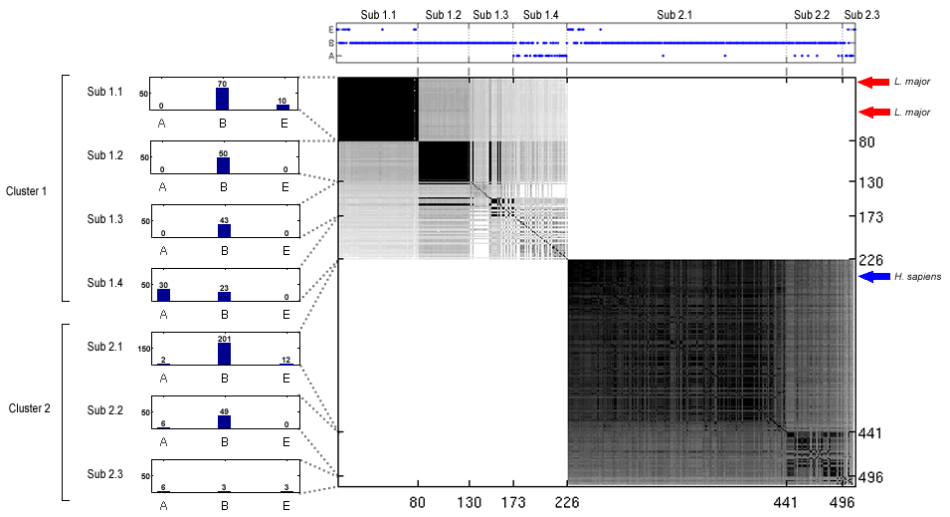
**Similarity matrix**. Similarity matrix (central figure) of EC 4.2.1.2 (fumarate hydratase). Each point of the 508 × 508 matrix represents the blastp similarity score of two enzymes. All scores above 1500 are reset to 1500. Higher similarity scores yield darker points, (white represents a score below 120). Sequences were sorted by the similarity score, using the longest enzymes as reference to the other enzymes. The three arrows on the right site indicate the positions of enzymes of *L. major *(red arrows) and *H. sapiens *(blue arrow). Histograms on the left display the distribution of organisms represented in each cluster, for the three kingdoms: archaebacteria (A), bacteria (B) and eukaryotes (E). In each of the two main (analogous) clusters, subclusters can be observed. The graph at the top of the matrix displays the kingdom of the organism for every enzyme in the matrix.

The clustering algorithm was implemented similarly to the method proposed by Galperin [7]. First, sequences with less than 100 amino acids were excluded from the dataset. For each enzymatic activity, an all-against-all BLASTp [3] (using a maximum e-value of 0.01 and standard parameters) was executed and results were transformed in a graph where each node represents an enzyme [14]. Two nodes are connected by an unweighted and undirected edge if they belong to the same EC class and have a similarity score higher or equal to 120 (an e-value close to *e*^-6^) [7]. This parameter (and others) can be modified by the user. All sequences connected in the graph were assigned to the same cluster and are considered homologous. Sequences not connected by a path in the graph are considered analogous. Therefore, the number of disconnected sub graphs would, in principle, represent the number of times that the enzymatic activity in question is thought to have appeared during evolution within the current dataset. As a representation of the graph, an adjacency-matrix [15] was implemented (Figure 2). Each cluster is finally stored in a flat file database.

### Filters for the datasets

Dataset a, the less conservative, is composed by all clusters formed after the initial clustering step. This dataset was further refined, using more stringent criteria. Filters were applied in four successive steps: Firstly, all clusters with only one sequence (singlets) were excluded (dataset B). Secondly, all enzymatic activities not defined up to the fourth level of the EC classification were also excluded (dataset C). Thirdly, all clusters of a determined function with proteins annotated as subunits of this function and Belonging to the same species were joined (dataset D). Finally, all clusters displaying putative intragenomic analogy (here defined as the identification of analogy between two enzymes in the same genome) were also joined (dataset E).

### Metabolic map reconstruction

Each result can be visualized as a metabolic map by using an external resource (a KEGG tool – [16]). Further, EC classes with potential cases of analogy or without representative sequences are highlighted. Color codes are used to discriminate the significance of the results, as well as the presence of analogy.

### Detection of analogy

In this work, we define a potential case of analogy if the sequences from a given enzymatic activity present in the genome of a single organism or between two organisms are placed in different clusters after grouping (intra-genomic and inter-genomic analogy, respectively). Orthologs or recently duplicated paralogs would be placed in the same cluster. Therefore AnEnPi compares, within a single species or between two species, the presence of a given function in each cluster, for all species currently represented in KEGG database. In the metabolic reconstruction step, the presence of a given enzymatic activity in a genome, the presence of analogy and the degree of significance of similarity searches can be highlighted. The result is an interactive list (in HTML or text format) with links to the EC classes and the metabolic maps.

### Annotation

For the purpose of annotation and identification, the user can perform similarity searches either by BLASTp or HMMSearch. In the first case, the database is composed of all proteins present in the clusters; in the second case, the database is composed of the probabilistic models constructed for each cluster, if the cluster has more than one element. For the construction of the latter, a multiple alignment was executed with ClustalW [17] and then transformed into a HMMer model with the functionalities available in the HMMer package. This type of annotation is based on the quality of previously annotated databases.

Therefore, we have introduced filters allowing the construction of different datasets, minimizing the number of wrongly identified cases of analogy and wrongly attributed functions. To our knowledge, AnEnPi is the only tool that provides annotation functionalities with emphasis on analogous enzymes.

### Front-end

All components described so far (Figure 1) were included in a user-friendly web-based interface named AnEnPi. All main functionalities are independent processes and may be used in different contexts, for instance in the identification of analogous enzymes, in sequence annotation, clustering or metabolic reconstruction. Also, sequences entered by the user can be clustered and converted into an annotation database for similarity searches. Results are displayed in a web page.

## Results

We implemented a web based interface  called AnEnPi (Analogous Enzyme Pipeline) that can be used for the annotation and visualization of metabolic pathways, and for the detection of events of analogy. As an example, we applied AnEnPi to identify possible cases of analogy between enzymes present in the genome of *L. major *[18] and the human genome, using a clusters From the KEGG database to form our reference datasets.

### Work flow, user interface, sequences and organisms

Clustering of the dataset obtained from KEGG produced 6,701 clusters, with 986 enzymatic activities (from the 2,433 represented in the KEGG dataset) having more than one cluster (approximately 40.5%) (Table 1). 2,199 sequences formed singlets, while 328 EC classes had more than three clusters. Table 1 shows the number of enzymatic activities with putative analogy before and after the four steps of data filtering. After the third filtering step (dataset D), the number of functions with more than 5 clusters drops to 46. Still, even after the application of these 3 filters, 19% of the enzymatic activities contain putative analogous sequences.

**Table 1:** Refinement of The initial Dataset (A) through the application of successive filters.

Datasets	# Clusters				Max. Clusters	% Analogous
	1	2	3	> 3		
A	1447	459	199	328	131	40.5
B	1600	345	113	180	78	26.2
C	1560	316	91	97	46	20.7
D	1619	302	73	70	23	19.4
E	1897	142	23	1	5	8.1

Table 1: A, dataset obtained after clustering; B, dataset obtained after the exclusion of singlets (clusters With only one sequence); C, dataset obtained after the exclusion of EC's which are not defined up to the Fourth level (incomplete EC' s); D, dataset obtained after the joining of clusters where some sequences Were annotated as 'subunits'; E, dataset obtained after the joining of clusters with putative intragenomic Analogy. Max. Cluster, the maximum number of clusters found for one specific enzymatic activity; % analogous, fraction of enzymatic activities where analogy was detected. # Clusters: number of functions with, respectively, 1, 2, 3 or more than 3 clusters.

### Comparison with literature data

To validate our results, we have searched the literature for known cases of analogy, predicted or confirmed through diverse approaches, such as computational and/or experimental methods. We compared our results with those of [7], where 108 cases of analogy were described. Only for three enzymatic activities the number of clusters produced by AnEnPi was smaller, in each case due to dataset differences. For all other cases we found at least the same number of clusters, demonstrating the consistency of the results. Table 2 illustrates some cases of analogy found in the literature. All functions listed also display potential cases of analogous sequences in our results, provided that the enzymatic activity in question is included in KEGG.

**Table 2:** Examples of analogy Found in The literature and the Methods used.

EC	Enzyme	Organism	Ref.	Method
1.1.1.42	Isocitrate dehydrogenase	*Escherichia coli*/*Azotobacter vinelandii*	[7]	a
2.7.1.4	Fructokinase	*Homo sapiens*/*Streptococcus mutans*	[7]	a
3.2.1.86	6-phospho-beta-glucosidase	*E. coli*/*H. sapiens*	[7]	a
3.4.21.72	Immunoglobulin A (IgA) proteases	*Streptococcus sanguis*/*Neisseria gonorhoeae*	[27]	a
2.1.1.-	N-methyltransferase 1	*Schizosaccharomyces pombe*/*Chlamydia pneumoniae*/*Archaeoglobus fulgidus*	[9]	a, b
2.7.7.-	Adenylyltransferase	*Bacillus subtilis*/*Saccharomyces cerevisiae*	[9]	a, b
3.1.3.11	Fructose-1,6-bisphosphatase	*Prochlorococcus marinus*/*E. coli*	[28]	c
2.3.1.-	Enoyl thioester reductase	*E. coli*/Yeast/Rat	[29]	d
1.13.11.2	Catechol 2,3-dioxygenase	*Pseudomonas sp*./TOL plasmid (pww0)	[30]	e
2.7.2.3	Glyceric acid 3-phosphate kinase	*Pisum sativum*	[31]	f
5.3.1.1	Triose phosphate isomerase	*Pisum sativum*	[31]	f

Table 2: a – computational, b – genetic complementation, c – genome sequencing, d – stereochemical assay, e – biochemical assay and f – isoelectric focusing

### Adjacency matrices and kingdom line

Figure 2 shows an adjacency-matrix for EC 4.2.1.2.(fumarate hydratase), a representation of an all-against-all BLASTp of all proteins belonging to this enzymatic activity. Each point in the matrix indicates the similarity score between two enzymes. Two main clusters can be seen, where sequences from one cluster have no detectable similarity with sequences from the other cluster. Some sub-clusters can be seen inside each main cluster, representing groups of more similar sequences, particularly inside cluster 1.

The histograms on the left and the 'kingdom line' above the matrix show the distribution of the organisms represented in the matrix in terms of kingdoms. Sub-cluster 1.1 has sequences derived from eukaryotes and eubacteria, while sub-clusters 1.2 and 1.3 from eubacteria only. The remaining sequences of cluster 1 do not form a well-defined sub-cluster, but archaeabacterial sequences are present only in this structure.

However, further identification of subclusters will be studied in another work, as a refinement of the present methodology. As an example of a potential case of analogy, for this EC function the location of the corresponding enzymes of *L. major *(two genes) and *H. sapiens *are displayed.

### Analogy identification

**Figure 3:**
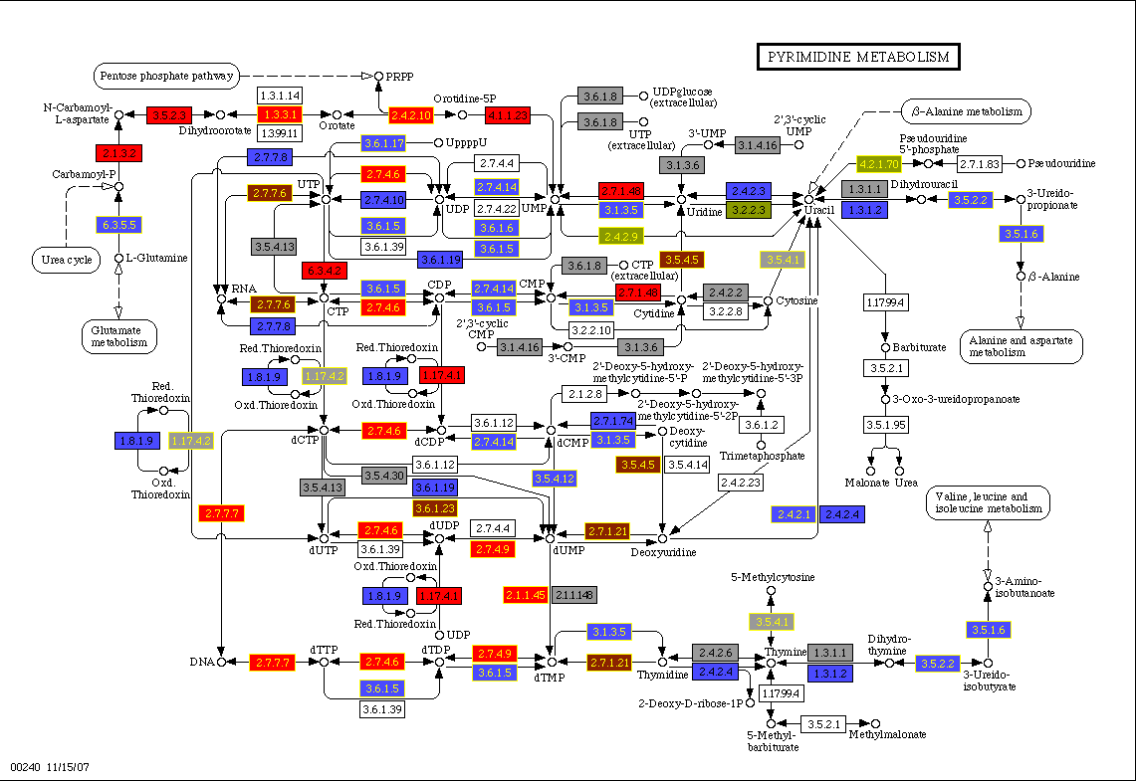
**Comparative analysis between H. sapiens and L. major**. Brown: analogy between two genes with the same function; red: function present in both species; blue: Function present only in *H. sapiens*; green: function present only in *L. major*; gray: function not found in both species and white: no representative enzymes in KEGG.

When applying AnEnPi to find analogy between *H. sapiens *and *L. major*, thirty-five potential cases of analogy were found using dataset D (Table 1). In twelve cases (EC 1.1.1.2, EC 1.3.1.34, EC 1.3.3.4, EC 2.3.1.48, EC 2.7.1.2, EC 2.7.4.2, EC 3.5.1.14, EC 3.6.1.23, EC 4.2.1.1, EC 4.2.1.2 (Figure 2), EC 5.3.1.6 and EC 5.3.3.2), inter-genomic analogy was found. The smallest cluster found comprised 8 individual protein sequences. The great majority (80 well as intra-genomic analogy. For example, in EC 2.1.1.17 (phosphatidylethanolamine N-methyltransferase), enzymes of *L. major *and *H. sapiens *share the cluster #4, but enzymes of *L. major *were also found in cluster #3. Therefore, *L. major *enzymes from cluster #3 are analogous to *H. sapiens *sequences present in cluster #4, and the sequences from both organisms present in cluster #4 are homologous. This function can also be used to display any other differences between two species in the web frontend (Figure 3).

### Intragenomic analogy

As described above, EC 2.1.1.17 is also a case of intragenomic analogy in *L. major*. With AnEnPi we detected a total of 12 cases of intragenomic analogy in *L. major *using dataset D. Application of AnEnPi to Datasets A, B and C returned 34, 34 and 23 cases, respectively. No intragenomic analogy is detected when using dataset e, because all clusters (from a particular enzymatic activity) with sequences from the same species are joined. These cases of intragenomic analogy were not related to a particular metabolic pathway.

One example of intragenomic analogy can be seen in the fructose and mannose metabolism (KEGG map 00051), where two unrelated sequences of phosphomannomutase (EC 5.4.2.8) were found in the *L. major *genome. A bibliographic search revealed almost no data besides the identification of these twelve enzymatic Activities in *L. major*, neither were we able to find systematic studies of intragenomic analogy in general.

## Discussion

We described in this work AnEnPi, a tool that can be used for the annotation and detection of analogous enzymes [19], improving the understanding of the biochemical pathways of the species under analysis. It offers functionalities for clustering, annotation, or pairwise comparisons between different species, intended for the identification and improvement of annotation of putative analogous enzymes.

Other tools like KAAS [20] or KOBAS [21] also perform whole genome annotation of enzymes, but AnEnPi is unique in the detection, comparison and visualization of events of analogy. In the advanced parameter settings, each threshold can be modified, such as for clustering, which should be of use for a large group of users.

The identification of structurally unrelated enzymes sharing the same enzymatic activity may reveal new catalytic mechanisms, lead to studies on the origin and evolution of biochemical systems and pathways, and also provide new candidates for drug design and development [22]. AnEnPi is an implementation of a methodology designed to help in the identification and annotation of putative events of independent origin of enzymatic activities through the clustering of their primary sequences [23]. AnEnPi also provides information for a more detailed reconstruction of metabolic pathways, including the significance of similarity scores and the presence/absence of alternative forms of a given enzymatic activity.

It is not a simple task to determine if two different proteins are derived from the same ancestor. Two homologous proteins may lack major sequence similarity and yet share a common origin, for example after many years of evolution [8]. The cut-off used in this work, a similarity score of 120, is based on the observation that there is a lack of similarity between the tertiary structures of proteins below this value [7].

Still, it is possible that two enzymes assigned by AnEnPi as analogous are in fact derived from the same ancestor but have diverged up to a point where their primary sequences no longer share recognizable similarity. Molecular modeling techniques, together with appropriate evolutionary methods, could be used to ascertain that the tri-dimensional structures and sequences of the enzymes assigned as analogous are indeed different, suggesting their independent origin.

To overcome some of these difficulties, methods to deduce functional information from a certain gene in the absence of sequence data have been recently proposed [24]. Needless to say, most approaches rely on high-quality annotation. As a matter of fact, problems with the data structure of some databases may create undesirable biases in our analyses. For instance, we have observed that annotation for a specific enzymatic activity for one particular subunit of a multimeric enzyme is commonly 'inherited' by all other subunits composing that enzyme. If these subunits are encoded by unrelated genes and do not have the same function, false cases of analogy will be computed. False cases of analogy will also appear for enzymatic activities that are dependent on the simultaneous presence of more than one type of subunit to form the catalytic site. In other words, if a hetero-multimeric enzyme is composed of subunits with different origins, AnEnPi may interpret the lack of similarity between said subunits as another case of analogy.

Although we have so far no automatic way to further refine our dataset, the distribution pattern of species over the clusters of a given enzymatic activity may indicate the presence of false positives and therefore serve as a criterion for their identification: the presence of representatives (proteins) from the same organism in several clusters would mean that that organism has several unrelated enzymes able to fulfill the same metabolic step. While this may be real, it is likely that a substantial part of these events are indeed annotation artifacts. In general, our results were congruent with the available literature on the subject (Table 2).

It is thus important to discriminate between i) two (or more) subunits of a given heteromultimeric enzyme encoded by unrelated genes and ii) two (or more) enzymes actually sharing the same function, also encoded by unrelated genes. Table 1 displays the results found when applying these criteria to improve the dataset.

Most likely, the majority of the clusters with only one representative sequence are possibly cases derived from wrong annotations or cases of very divergent sequences, which are not included in other clusters due to the cut-off used. As an example, analysis of *T. brucei *data produced 14 singlets. The annotation of the metabolic pathways in this organism was done manually [25], and results entered in the KEGG database.

The user should choose the best dataset for his purposes. To be conservative, we have employed in most of our analyses the dataset D, minimizing the number of false positives (and consequently probably loosing other real cases of analogy). Using the dataset E, though very restrictive and probably an underestimation, we obtained a set of analogies with a higher probability of being true cases, without possible errors due to multimeric proteins; in fact, even after applying all these criteria for data filtering, still 8.6% of all enzyme classes have potential cases of analogy. No doubt, a better handling of inconsistencies generated during the annotation of multimeric enzymes would improve the identification and provide a better estimation of the frequency and distribution of the cases of intragenomic analogy.

The ability to identify potential cases of analogy between genes from two different species (Figure 3), as well as differences in assigned functions, can be used to indicate the possibility of alternative pathways or disclose candidates for drug development. One example analyzed in more detail is fumarate hydratase (EC 4.2.1.2.) from *H. sapiens *and *L. major*, whose sequences were assigned to distinct clusters. AnEnPi can help by producing a list of shared enzymatic activities between the two organisms without detectable similarity at their primary level, reflecting substantial differences between their folding patterns. Also, the overall pattern of similarity scores shown in Figure 2 suggests that fumarate hydratase is evolving in distinct ways, depending on the group of organisms in question. More detailed studies are underway to investigate these points.

Currently, we are developing a database with all putative analogy events stored in a comprehensive way, linked to information from drug databases. AnEnPi is also being updated, with the inclusion of information from hundreds of new organisms.

## Conclusion

AnEnPi is a versatile tool designed to assist the user in the identification, clustering and annotation of analogous enzymes. Its modular structure allows its utilization in other contexts. Addition of color codes to represent biological attributes allows for a better visualization of metabolic pathways, with more meaningful biological information, facilitating the interpretation of the results.

## 1 Availability and requirements

AnEnPi is freely accessible at .

• Project name: AnEnPi – Analogous Enzyme Pipeline (Webserver)

• Project home page: 

• Operating system: Linux

• Programming language: Perl and HTML

• Licence: AnEnPi is accessible under a GPL license

## 2 Abbreviations

EC: enzyme commission; AnEnPi: Analogous Enzyme Pipeline

## Authors' contributions

The tool was developed and implemented by TO. He and AG designed the web interface and carried out the experiments. All authors analyzed together the data, discussed the results, wrote the manuscript and approved the final version.
